# Surface Roughening of Irradiation-Activated Basalt Fiber through In Situ Growth of SiO_2_: Effects on Crystallization and Properties of PP Composites

**DOI:** 10.3390/ma16165657

**Published:** 2023-08-17

**Authors:** Shuai Zhang, Pan He, Shuoyi Jing, Gang Chen, Guangzhao Li, Zhongzui Wang, Rui Han, Yijun Li

**Affiliations:** 1 School of Materials Science and Engineering, Xihua University, Chengdu 610039, China; brucezhang.scu@foxmail.com (S.Z.); gangchen@mail.xhu.edu.cn (G.C.); guangzhao.li@hotmail.com (G.L.); wzz0901@163.com (Z.W.); 2 Engineering Research Center of Intelligent Air-Ground Integration Vehicle and Control, Ministry of Education, Xihua University, Chengdu 610039, China; 3 Sichuan Provincial Engineering Research Center of Functional Development and Application of High Performance Special Textile Materials, Chengdu Textile College, Chengdu 611731, China; hepan-2009@163.com; 4 Sichuan Special Equipment Inspection Institute, Chengdu 610100, China; jingshuoyi@scsei.org.cn; 5 Technology Innovation Center of Hydrogen Storage-Transportation and Fueling Equipments for State Market Regulation, Chengdu 610100, China; 6 State Key Laboratory of Polymer Materials Engineering, Polymer Research Institute, Sichuan University, Chengdu 610065, China

**Keywords:** basalt fiber, electron beam irradiation, surface roughening, polypropylene, macro-performance

## Abstract

Basalt fiber (BF) is deemed a new environmentally friendly and high-performance fiber material due to its high strength, electrical insulation, corrosion resistance and high temperature resistance. Yet, the surface inertness restricts its practical application. In this work, the BF was irradiated and activated by electron beam, followed by in situ growth of SiO_2_ using a hydrothermal method, then composites with polypropylene (PP) were prepared by microinjection molding. According to the results of scanning electron microscopy (SEM) and Fourier transform infrared (FTIR), more active sites can be formed after irradiation, thus more SiO_2_ nanoparticles were generated on the surface of BF. Consequently, the rough surface of modified BF could provide stronger shear force during melt processing and resulted in a higher orientation of the molecular chains, increasing the lamellar thickness and generating more highly ordered β crystals in the composites. I_400_BF-gSiO_2_ exhibited the highest content of β crystals with the crystallinity of 53.62% and orientation of β (300) crystal plane of 0.91, which were 8.66% and 0.04 higher than those of the composite with pristine BF. Furthermore, due to the perfection of crystals, increased interfaces and interfacial interlocking between PP molecules and modified BF, I_400_BF-gSiO_2_ showed good overall performance, with storage modulus of 8000 MPa at −100 °C, glass transition temperature of 23.03 °C and tensile strength of 62.2 MPa, which was 1900 MPa, 1.23 °C and 29.6 MPa higher than neat PP. Hence, the surface roughing strategy proposed in this work is expected to provide some insight and promote the application of BF reinforced thermoplastic composites.

## 1. Introduction

Fiber-reinforced polymer composites play a key role in our life, which are indispensable materials in many fields, for example, automobiles, ships, rail traffics, aerospace, medical apparatuses and military equipment. Various fibers can be used as reinforcements, such as glass fiber, carbon fiber, Kevlar, polyester fiber and all kinds of natural fibers [1,2,3]. Natural fibers such as kenaf and flax are eco-friendly and renewable, but the mechanical properties of these are unsatisfying and can be easily degraded by heat [4,5,6]. Most synthetic polymer fibers can only be used in mild environments due to their poor thermal resistance. Kevlar and carbon fiber can withstand high temperature application, but perform poor oxidation resistance. As a relatively new type of continuous inorganic fiber, basalt fiber (BF) not only shows higher thermal and oxidation resistance than organic fibers and carbon fiber, but also possesses higher mechanical strength than that of glass fibers. Additionally, the BF is prepared by rapidly drawing the basalt melt through the platinum–rhodium alloy wire drawing leakage plate at 1450 °C~1500 °C [7,8,9], the production process of BF produces little waste and pollution, hence it is deemed as a green and environmentally friendly fiber [10,11,12,13]. After years of academic research, industrial investment and market promotion, BF has shown great potential in structural materials, construction materials, friction materials, shipbuilding materials, heat insulation materials, automotive industry, high-temperature filter fabrics and protection fields [14,15,16,17,18,19,20].

Though BF possesses many merits, the main component of BF, metallic oxides, causes its surface to be inert, limiting its wide application. Furthermore, the advantages of the BF cannot be made good use of due to the poor interface interaction between the fiber and the polymer matrix. Hence, many efforts have been made to tackle this issue [21] and many BF-based composites with thermoplastic polymers have been developed. For example, Bin Wei [22] explored the nanomaterial-reinforced polymer coating on BF and found that when the polymer coating containing 5 wt% of nanoparticles was applied to the surface of BF, the tensile strength of the composites was significantly improved. Cagrialp Arslan [23] coated the BF with three different silane coupling agents and prepared composites with poly (butyleneterefthalate) (PBT), which showed that the covalent bond formed between BF and PBT caused an improvement in mechanical properties including tensile and flexural strength and elastic modulus [24,25]. Additionally, etching of BF is able to produce rough surfaces and increase interfaces, but the mechanical property of BF is greatly hampered [22,26,27,28]. Therefore, it is believed that the modification of BF to produce rough surface or increase the chemical reactivity without reducing the physical integrity of BF is the key point in making the most of BF. Surface roughing is believed to enhance the physical interlock between the surface of fiber and the polymer chain and increase the thickness of the interface, thus the strong fiber can better bear the force and prevent chain slippage and plastic deformation [29,30,31,32]. 

Though an improvement in mechanical properties can be achieved by simply adding nanoparticles or coating with silane coupling agents, it is of great value to investigate the effect of both chemical bond formation and surface roughing. Electron beam irradiation modification is an effective way to enhance the surface chemical reactivity of the fibers without affecting the integrity, hence it has been broadly used to modify organic fibers such as polyacrylonitrile and polypropylene [33,34,35]. However, the irradiation modification of BF is scarcely reported. It is anticipated that, after irradiation, the formation of active sites may make the BF chemical modification or surface roughing easier. Furthermore, by taking advantage of the active sites, SiO_2_ nanoparticles may grow on the surface of BF, the chemical bonds formed between SiO_2_ nanoparticles and BF may ensure strong adhesion of SiO_2_ nanoparticles and maximize the enhancement of the modified BF. Polypropylene (PP) is used as the polymer matrix here because it is one of the most widely used thermoplastic polymers. And its inherent disadvantages, such as relatively low mechanical properties, high shrinkage, ease of melting, inflammability and poor aging resistance may be improved by the introduction of BF [36,37,38,39,40]. Therefore, in order to probe the reinforcing effect of both the chemical modification and introduction of nanoparticles, we used electron beam to activate the BF and SiO_2_ nanoparticles were grown in situ on the surface of BF, then the PP composites were prepared. The effect of surface-roughed BF on the crystallization and properties of the composites were systematically studied.

## 2. Experimental

### 2.1. Materials

Isotatic polypropylene (PP) T30s, white granules with isotacticity of 98% and melting point of 165.4 °C, was purchased from Lanzhou Petrochemical Company (Lanzhou, China). Basalt fiber (BF), with average length around 6 mm, diameter around 17 μm, density of 2.65 g/cm^3^, elasticity modulus of 7.6 GPa and tensile strength of 1050 MPa, was purchased from Changsha Huixiang Building Materials Co., Ltd. (Changsha, China) Absolute ethyl alcohol (AR grade, >99.5%), deionized water, tetraethoxysilane (AR grade, SiO_2_ > 28%), acrylic acid (AR grade, >99.5%) and ammonium hydroxide (AR grade, 25–28%) were all purchased from Chengdu Kelong Chemical Reagents Co., Ltd. (Chengdu, China) and used without any further treatment.

### 2.2. Irradiation Treatment of BF and In Situ Growth of SiO_2_ Nanoparticles

**Scheme 1:**
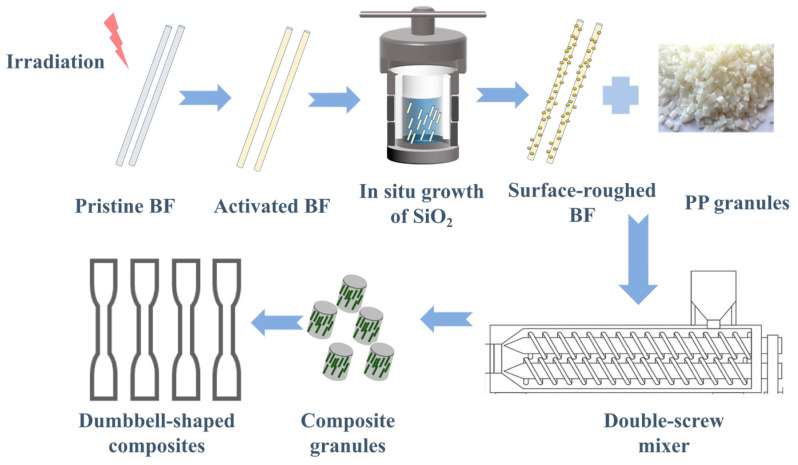
Preparation path for surface-roughed BF and BP/PP composites.

The pristine BF was firstly treated at 400 °C for 15 min to remove impurities. Then, as Scheme 1 shows, the BF was treated by GJ-2 electron accelerator in air with irradiation dose of 100 KGy, 200 KGy, 400 KGy and 800 KGy, respectively. The GJ-2 electron accelerator is an industrial grade accelerator made by Shanghai Xianfeng motor factory, with maximum power of 20 MeV, electron beam of 10 mA and scanning width of 1000 mm. The growth of SiO_2_ was referred to in a previous work [21]. Specifically, in a 500 mL beaker, 100 mL of absolute ethyl alcohol and 11.8 mL of ammonium hydroxide were added and stirred at room temperature for 10 min, followed by the slow addition of 6.2 mL of tetraethoxysilane. The mixture was then stirred for 6 h with the agitator speed of 200 rpm. Afterwards, 6 g of irradiation-treated BF was added and stirred for 30 min, followed by ultrasonic treatment for 20 min. Then, the mixture was decanted into an autoclave and placed in an oven at 140 °C for 3 h. After cooling, the precipitates were washed by ethyl alcohol and water 3 times, respectively, and then dried at 80 °C for 6–8 h. The obtained products were named as I_0_BF-gSiO_2_, I_100_BF-gSiO_2_, I_200_BF-gSiO_2_, I_400_BF-gSiO_2_ and I_800_BF-gSiO_2_, according to the irradiation dose applied, and the subscripted number represents the dosage.

### 2.3. Preparation of BF/PP Composites

As shown in Scheme 1 and referred to in our previous work [41], PP granules were dried in an air-circulating oven at 80 °C for 4 h, then PP granules and 20 wt% of BF were blended in a TSE-30A corotating double-screw extruder (Nanjing Rayavast Polymer Equipment Co., Ltd., Nanjing, China) at 190 °C for 10 min using rotation speed of 25 r/min. The dumbbell-like samples were prepared by a micro injection molding machine with temperature of 190 °C and injection rate of 100 mm/s. 

### 2.4. Characterization

Attenuated total reflection infrared (FTIR-ATR) spectra were obtained using a Nicolet-560 spectrometer (Thermo Nicolet Corporation, Madison, WI, USA) in the range of 4000–500 cm^−1^ at a resolution of 4 cm^−1^. Philips XL-30 FEG (Royal Philips Netherlands, Amsterdam, The Netherlands) scanning electron microscope was used to observe the surface of BP and the fracture sections of the composites; all test samples were coated with ultrathin electricity-conducting gold by high vacuum evaporation before observation. Differential scanning calorimetry (DSC) tests were conducted using a DSC Q25 (TA Instruments, New Castle, DE, USA) at a heating rate of 10 °C/min under nitrogen atmosphere. Calibration was performed using an indium standard and about 3 mg of samples were sealed in the aluminum pan for scanning. The first ramping and the first cooling was recorded in order to reflect the real character of the samples. Thermal gravimetric analysis (TGA) was performed on TGA 4000 (Perkin Elmer, Waltham, MA, USA) from 40 °C to 800 °C in nitrogen atmosphere with a flow rate of 40 mL/min and a heating rate of 10 °C/min. The phase analysis of the samples was performed by using an X-ray diffraction (XRD, smartlab, Rigaku Corporation, Osaka, Japan) with Cu target operating at 45 kV. The scanning range was from 2θ = 10–40° at a scanning rate of 0.01 °/s. Both two-dimensional wide-angle X-ray diffraction (2D-WAXD) and two-dimensional small-angle X-ray scattering (2D-SAXS) were performed at the Shanghai synchrotron radiation source time resolution ultra small-angle scattering line station BL10U1. The distance from the tested sample to the wide-angle X-ray diffraction detector was set as 70 mm, the distance from the tested sample to the small-angle X-ray scattering detector was set as 2495 mm and the exposure time was 20 s. The 2D-WAXD and 2D-SAXS characterization were conducted at the same time so the whole 2D-WAXD patterns cannot be shown. Dynamic mechanical analysis (DMA) was performed on a DMA Q800 dynamic mechanical analyzer (TA Instruments) with a frequency of 1 Hz and a heating rate of 5 °C/min, from −100 °C to 150 °C in air atmosphere using tensile mode. The tensile tests were conducted on an INSTRON 5967 universal testing machine following the ASTM D882 with a tensile speed of 20 mm/min, all the reported data were the average of at least five tests. The size of the central part of the dumbbell sample was round 10 mm × 2 mm × 0.3 mm. The relatively low tensile speed was chosen for better observation of the yielding behavior. A FOTRIC thermal imager was used to capture the infrared thermal imaging of the samples on a thermostatic heating plate, in order to probe the heat-insulating properties of the composites.

## 3. Results and Discussion

### 3.1. Characterization of BF with In Situ Growth of SiO_2_ Nanoparticles

**Figure 1:**
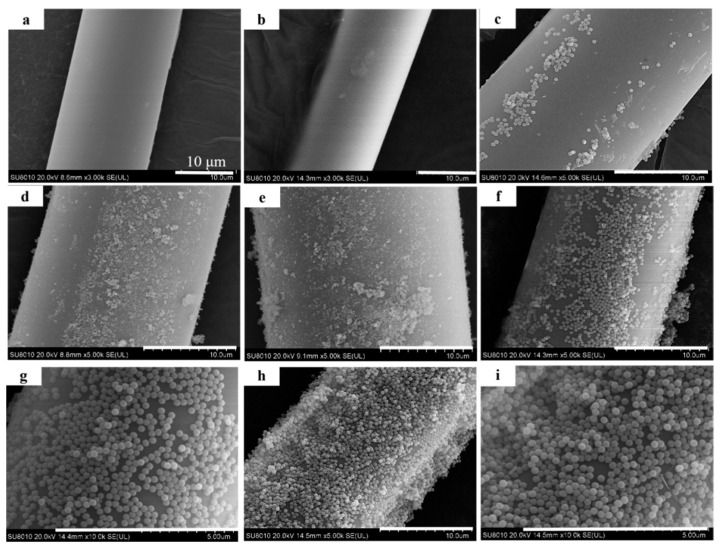
SEM images of BF (**a**), BF after irradiation treatment of 400 KGy (**b**), I_0_BF-gSiO_2_ (**c**), I_100_BF-gSiO_2_ (**d**), I_200_BF-gSiO_2_ (**e**), I_400_BF-gSiO_2_ (**f**,**g**) and I_800_BF-gSiO_2_ (**h**,**i**). The white scale bars represent length of 10 μm.

To verify the successful modification of BF by the in situ growth of SiO_2_ nanoparticles, SEM images of the fibers were obtained and shown in Figure 1. As shown in Figure 1a, the surface of the neat BF was quite smooth. After irradiation, the surface of the fiber showed little physical change (Figure 1b), which may be attributed to the fact that BF is quite stable and inert. In addition, the variation of chemical bonds cannot be detected by morphology observation. After the growth of SiO_2_, some sphere particles at a submicron scale can be seen on the surface of BF without irradiation treatment (Figure 1c); the amount of the SiO_2_ spheres was few and the distribution of the SiO_2_ spheres was uneven. For BF treated with relatively low radiation dose (100 and 200 KGy), flake-lie SiO_2_ can be observed on the whole surface of the BF, and the amount increased with higher radiation dose (Figure 1d,e). Such features may suggest that the irradiation treatment could activate the BF and generate some functional groups. With 400 KGy of electron beam irradiation, most of the BF surface was covered with SiO_2_ nanoparticles (Figure 1f,g), the majority of the SiO_2_ nanoparticles were distributed in a one-layer manner, but agglomeration and multi-layer SiO_2_ nanoparticles can also be seen. However, for I_800_BF-gSiO_2_ (Figure 1h,i), the surface of the BF was completely covered by multi-layer SiO_2_ nanoparticles. Hence, it can be concluded that electron beam irradiation would benefit the generation of SiO_2_ nanoparticles on the surface of BF.

**Figure 2:**
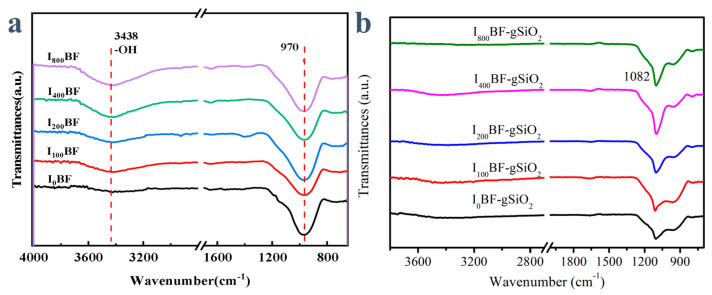
FTIR spectra of BF after irradiation treatment (**a**) and BF after in situ growth of SiO_2_ (**b**).

FTIR spectra of the fibers were obtained to further investigate the variation of chemical structures. As Figure 2a showed, with the increasing dose of irradiation, the peak intensity of hydroxyl groups centered at around 3438 cm^−1^ gradually increased, which means that the electron beam irradiation could indeed activate the BF and generate hydroxyls on the surface of the BF. The strong and wide band centered at 970 cm^−1^ was attributed to the vibration of Si-O bonds of aluminosilicate in BF. After the in situ growth of the SiO_2_ nanoparticles (Figure 2a), the peak of hydroxyl groups centered at around 3438 cm^−1^ disappeared almost completely. It was deduced that the hydroxyl groups may be consumed and form new chemical bond during the growth of the SiO_2_. Additionally, the successful growth of SiO_2_ can be proved by the emergence of the new band attributed to Si-O-Si stretching at around 1082 cm^−1^. When comparing the relative intensity of the peaks at 970 cm^−1^ and 1082 cm^−1^, it was found that the intensity of the peak centered at 1082 cm^−1^ increased gradually with the increasing dosage of irradiation, suggesting the generation of more SiO_2_ nanoparticles, which was consistent with the SEM observations. 

### 3.2. Crystal Structure of the BF/PP Composites

**Figure 3:**
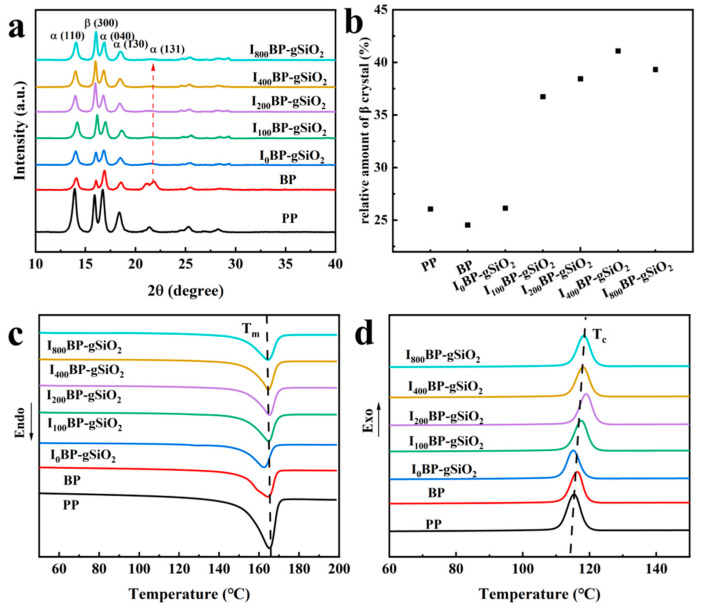
XRD patterns of the composites (**a**), relative amount of β crystal (**b**), DSC thermograms of the composites during heating (**c**) and cooling (**d**).

X-ray diffraction, as a nondestructive testing method, was firstly applied to probe the crystal structure of the composites. Three diffraction peaks were observed at 2θ = 14°, 16° and 18.6° corresponding to the α (110), α (040) and α (130) lattice plane, and one diffraction peak at 2θ = 16.8° corresponding to the β (300) crystal lattice plane. It is obvious that PP and BP showed relatively low intensity of the β (300) crystal lattice plane and then the intensity of the β (300) crystal lattice plane gradually increased for the other samples. The exact value of relative content of β crystal (*K*_β_) can be calculated according to Formula (1) [41,42].

(1) $$K_{\beta }=\frac{H(300)}{H(300)+H(110)+H(040)+H(130)}$$

where *H* (110), *H* (040) and *H* (130) represent the diffraction peak height of the α-crystal plane, respectively, while *H* (300) is the diffraction peak height of the β-crystal plane. As Figure 3d shows, neat PP possessed *K*_β_ around 25.8%, while BP showed the lowest value of 24.2% and I_400_BF-gSiO_2_ showed the highest value of 41.1%. The drastic increase in *K*_β_ may be caused by the heterogeneous nucleation effect induced by SiO_2_ nanoparticles, that is, SiO_2_ may function as a β nucleating agent that promotes the formation of β crystals by surface epiphysis [43,44]. As for I_800_BF-gSiO_2_, though the content of SiO_2_ nanoparticles was high, there were too many SiO_2_ nanoparticles and the outer layer of SiO_2_ nanoparticles may not be stable to withstand the micro-injection molding, hence the K_β_ slightly decreased.

The melting and crystallization behaviors of the composites were then studied using DSC and the results are shown in Figure 3 and Table 1. As shown in Figure 3a, almost all samples showed a single melting temperature (T_m_) in the range of 164–165 °C, except for I_0_BF-gSiO_2_. It should be noted that DSC testing is a dynamic process and the heating rate is too fast to distinguish the subtle crystal change. And β-crystal of PP is thermodynamically metastable, and transformation from β-crystal to α-crystal may happen easily during heating [44,45]. The transformation from metastable to steady crystal was an exothermic process, which was counteracted with the endothermic signal of crystal melting. Moreover, the peak width at half height (FWHM) of T_m_ decreased with the addition of SiO_2_-modified BF. I_400_BF-gSiO_2_ showed the smallest FWHM of 8.16 °C, 2.21 °C smaller than that of neat PP. Additionally, the melting enthalpy (∆H_m_) decreased and then increased with the addition of SiO_2_-modified BF and I_400_BF-gSiO_2_ showed the highest ∆H_m_ of 89.66 J/g. Hence, based on the features mentioned above, it can be concluded that the SiO_2_-modified BF could promote the crystallization of PP. As for I_800_BF-gSiO_2_, the lower T_m_, bigger FWHM and lower ∆H_m_ may be caused by the falling of massive and vulnerable SiO_2_ particles into the PP matrix that disturbed the crystallization of PP. Moreover, as Figure 3b shows, the crystallization temperature (T_c_) also increased gradually with the addition of SiO_2_-modified BF, I_200_BF-gSiO_2_ showed the highest T_c_ of 114.31 °C, which is 7.28 °C higher than that of neat PP while the T_c_s of I_200_BF-gSiO_2_, I_400_BF-gSiO_2_ and I_800_BF-gSiO_2_ were very close. The T_c_ is believed to be closely related to the amount of SiO_2_ nanoparticles formed, that is, more SiO_2_ would lead to higher T_c_ because SiO_2_ nanoparticles could act as heterogeneous nucleating agents for PP [44,45]. As is well known, the polymer chain would possess higher mobility with respect to higher T_c_. In addition, heterogeneous nucleation reduces the barrier of nucleation and promotes the increase in nucleation density, which is also beneficial to a higher degree of crystallinity, hence the crystallinity of I_400_BF-gSiO_2_ and I_800_BF-gSiO_2_ were both higher than 50%. 

**Figure 4:**
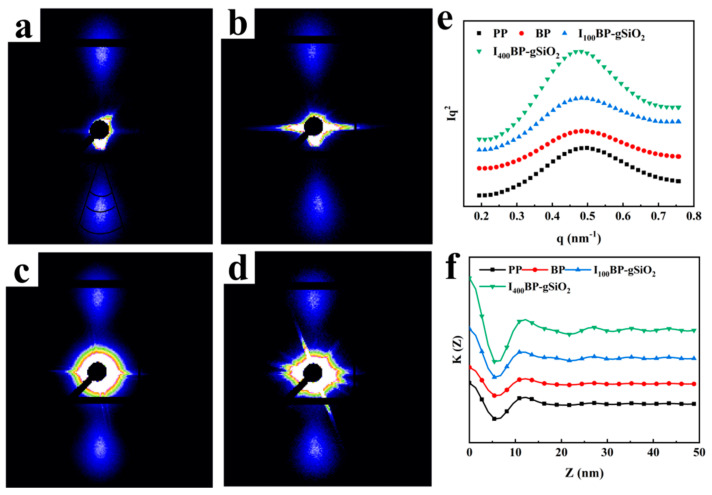
The 2D-SAXS patterns of the composites, neat PP (**a**), BP (**b**), I_100_BP-gSiO_2_ (**c**), I_400_BP-gSiO_2_ (**d**) and analysis of the samples, long period (**e**) and thickness of lamella and adjacent amorphous region (**f**).

In order to further analyze the crystal structure of the composites and elucidate the influence of surface roughness, the 2D small-angle diffraction (2D-SAXS) test was conducted and the results are shown in Figure 4a–d. The long period (*L*) of the oriented lamellar stacks was calculated through Bragg’s Law (Formula (2)), and the average lamellae thickness (D_c_) and the thickness of the amorphous region (D_a_) were determined by one-dimensional electron density correlation function (Formula (3)) [46,47,48].

(2) $$L=\frac{2\pi }{q_{max}}$$

(3) $$(z)=\frac{\int_{0}^{\infty }I(q)q^{2}\cos\left(qz\right)dq}{\int_{0}^{\infty }I(q)q^{2}dq}$$

where *q* = 4πsinθ/λ, 2θ is the scattering angle, λ is the wavelength, *q_max_* is the *q* corresponding to the maximum value of *I_q_*^2^, *Z* is the real space length parameter (nm) and *I*(*q*) is the one-dimensional curve density distribution of the two-dimensional scattering pattern. The data processing of 2D-SAXS was performed by Xpolar software for lamellae thickness. 

Accordingly, the L, D_c_ and D_a_ are summarized in Table 2. Neat PP showed the smallest L of 12.68 nm, while I_400_BF-gSiO_2_ showed the biggest L of 13.27 nm. Interestingly, I_400_BF-gSiO_2_ possessed reduced D_a_ of 4.98 nm, 0.03 nm smaller than that of PP, but the D_c_ of I_400_BF-gSiO_2_ reached 8.29 nm, 0.62 nm bigger than that of PP. Such results indicated that the introduction of roughed BF increased the long period size and the increase was mainly contributed by the crystalline lamella, which was consistent with the previous finding that the crystallinity increased with the introduction of modified BF. An important cause for such a phenomenon may be the heterogeneous nucleation that reduces the barrier of nucleation and promotes the increase in nucleation density and higher T_c_ that favored the rapid nuclei growth. 

**Figure 5:**
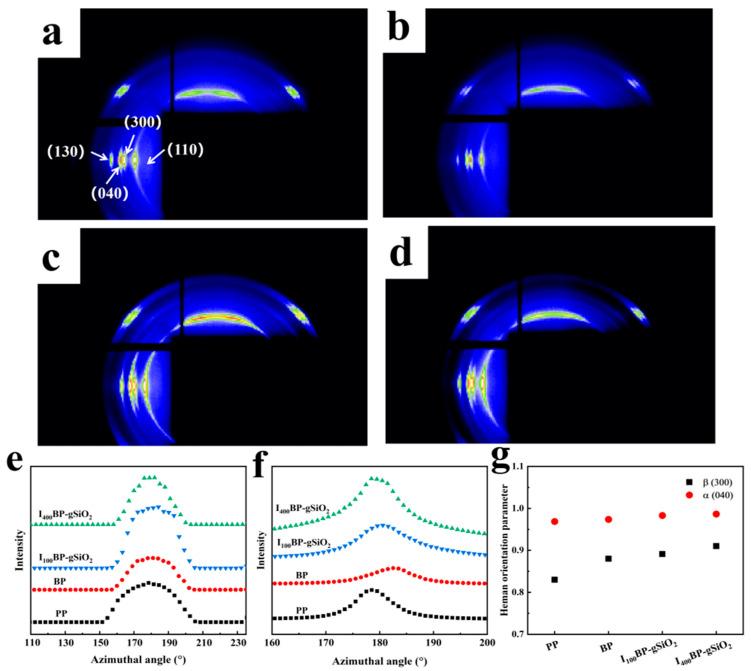
The 2D-WAXD patterns of the composites, neat PP (**a**), BP (**b**), I_100_BP-gSiO_2_ (**c**), I_400_BP-gSiO_2_ (**d**), the corresponding azimuthal intensity distribution of β (300) (**e**) and α (040) (**f**), lamellar orientation evaluated using Hermann orientation factor of the composites (**g**).

To investigate the lamella orientation of the composites, their 2D-WAXD patterns were presented in Figure 5. Six arcs can be detected in the patterns from inside to outside for each sample, which can be assigned to α-(110), β-(300), α-(040), α-(130), β-(311)/α-(111) and α-(041), respectively. The discontinuity of the rings was caused by the high orientation of the crystal. To track the evolution of the crystal orientation, 1D azimuthal integration curves of the crystallographic plane β-(300) and α-(040) were shown in Figure 5e,f. By using the Hermans orientation parameter *f* [41,49], the orientation degree can be calculated from the α-(040) and β-(300) intensity distribution along the azimuthal angle ranging from 110° to 240° and 160° to 200°, respectively, and the results are presented in Figure 5g (Formula (4)).

(4) $$x=\left\{\frac{3\cos^{2}\Psi -1}{2}\right\}$$

The orientation of the α-form crystal increased slightly and that of the β-form crystal increased from 0.83 of PP to 0.91 of I_400_BF-gSiO_2_. Such variation may also be caused by the fact that the rough surface of BF increased friction and contact area between PP chains and BF, thus the polymer chain was more extended and oriented due to the stronger shear force and the modified BF limited the chain relaxation during the cooling and crystallization process. To sum up, the rough surface of modified BF could provide stronger shear force and resulted in a higher orientation of the molecular chain, increasing the lamellar thickness and generating more highly ordered β crystals in the composite.

### 3.3. Dynamic Mechanical Properties of the BF/PP Composites

**Figure 6:**
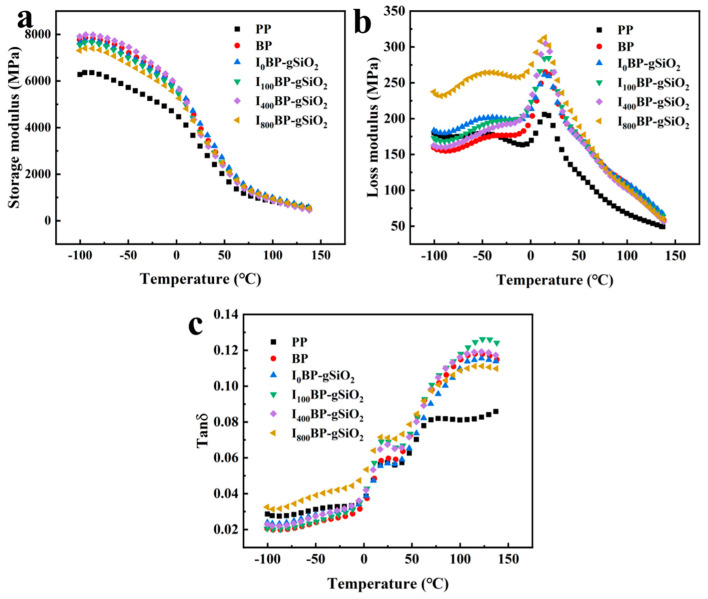
Storage modulus (**a**), loss modulus (**b**) and loss tangent curves (**c**) of the composites.

DMA is widely used to study the molecular relaxation process and the viscoelastic behavior of the polymers, and the interface interaction of the composites can also be reflected. Figure 6a showed the storage modulus, loss modulus and tangent loss thermograms of the composites. Neat PP possessed the lowest storage modulus around 6280 MPa at −100 °C, the introduction of BF increased the storage modulus to more than 7000 MPa, while I_400_BF-gSiO_2_ showed the highest storage modulus of 7909 MPa at −100 °C (Table 3). At 25 °C, however, I_0_BF-gSiO_2_ showed the highest storage modulus of 4136 MPa, while neat PP showed the lowest at 3180 MPa. The increased storage modulus was attributed to the reinforcing of rigid BF and indicated the strong interface interaction, which was believed to be caused by the entanglement of PP chains with SiO_2_ nanoparticles anchored on BF. When subjected to external forces, rough BF-reinforced PP composites can better bear the external loads during the material deformation process, which increases the storage modulus of the composite. Furthermore, the roughness BF can also occupy the space between the molecular chains of PP, increasing the friction between PP chains and restrict the movement of PP chains. 

As for glass transition temperature (T_g_), I_400_BF-gSiO_2_ also delivered the highest T_g_ of 23.03 °C, 1.23 °C higher than that of neat PP and 0.56 °C higher than that of BP. The improved thermal resistance may be correlated to the improved crystallinity and reinforcing of rigid BF. Specifically, higher crystallinity was beneficial for higher thermal resistance, and the surface-roughed BF reinforced the PP through strong interface interaction as well. 

### 3.4. Thermal Stability of the BF/PP Composites

**Figure 7:**
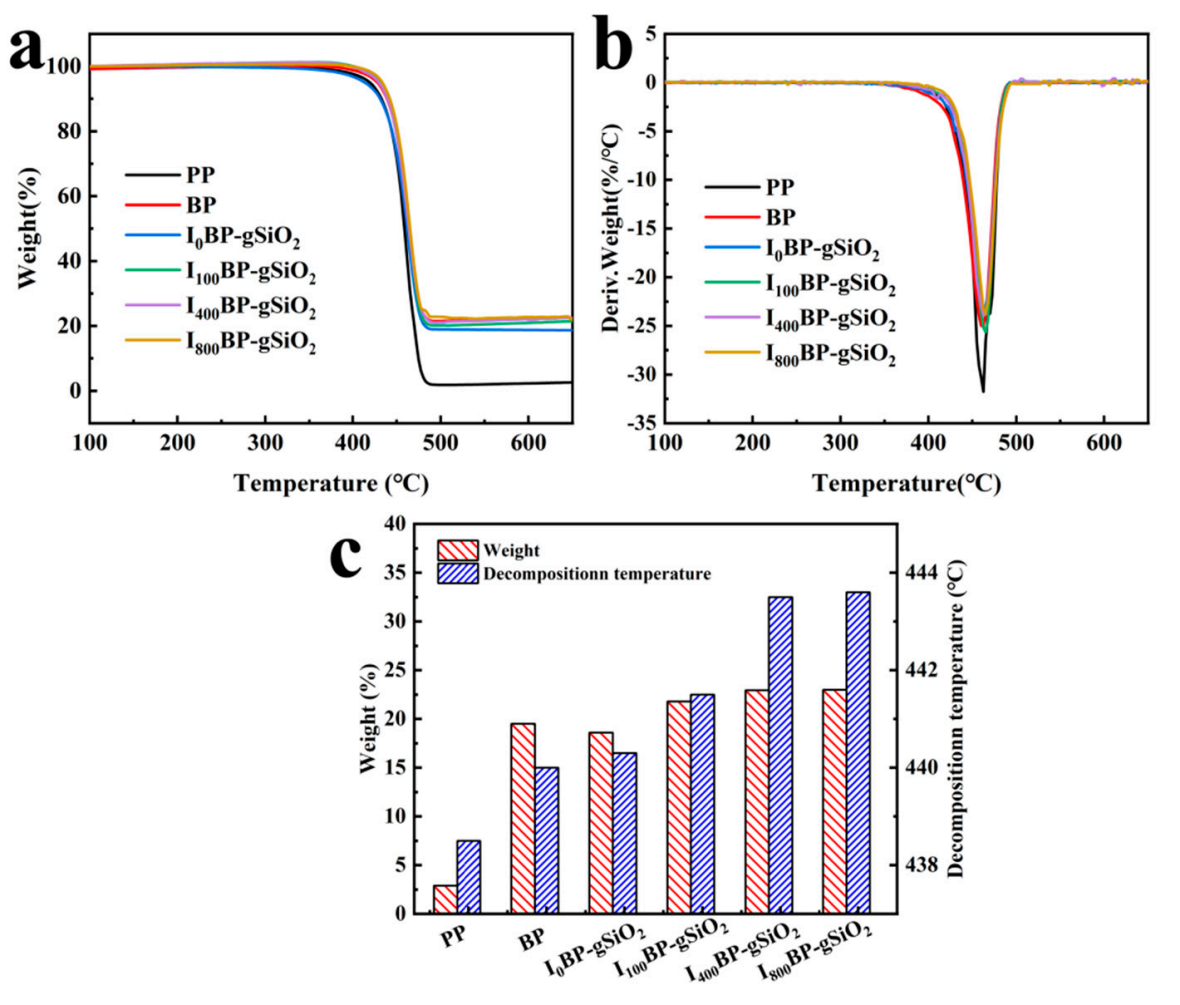
TGA (**a**), DTG (**b**) thermograms of the composites and summary of residual weight (**c**) and decomposition temperature (**c**).

Furthermore, the introduction of BF also enhanced the thermal stability of the composites. As Figure 7 shows, the highest initial decomposition temperature (T_i_) of 443.7 °C was reached by I_800_BF-gSiO_2_. Two reasons may be responsible for the improvement in T_i_: the intrinsically high thermal stability of BF and its barrier effect that slowed the chain scission.

### 3.5. Mechanical Properties of the BF/PP Composites

**Figure 8:**
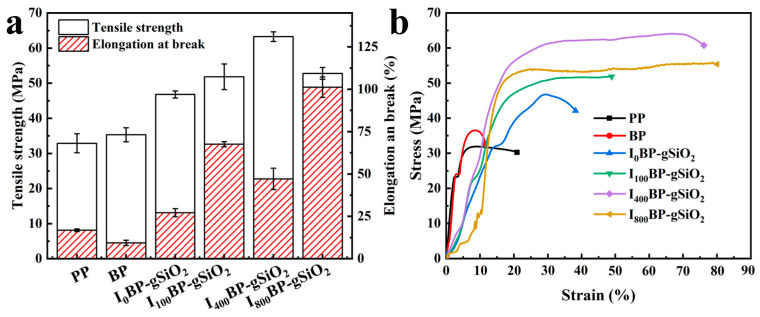
Tensile strength, elongation at break (**a**) and stress–strain curves (**b**) of the BF/PP composites.

To probe the effect of surface roughening and variation of crystal structure on the mechanical properties of the composites, tensile tests were carried out and the results are shown in Figure 8. It was learned that, with the modification of SiO_2_, the tensile strength and elongation at break value (EAB) increased with the higher dosage of electron beam irradiation. Neat PP showed a tensile strength and EAB of 32.6 MPa and 20.3%, while I_400_BF-gSiO_2_ showed the highest tensile strength of 62.2 MPa and improved EAB of 47.3%. Overall, when compared with the neat PP and BP, both mechanical strength and toughness were largely improved with the introduction of roughed BF. Such a result may originate from three reasons: first of all, the rigid BF, higher crystallinity, higher content of β crystal and higher orientation parameter were key to improved tensile strength; secondly, the strong interface interaction and chain entanglement would hinder the chain slipping; and lastly, the β crystal was intrinsically tougher than the α crystal because the β crystal was less dense and rigid. It was also noted that, when SiO_2_ was grown on the BF, the elastic modulus of the composites decreased slightly. Such a phenomenon seemed to be contradictory and may need further investigation. 

**Figure 9:**
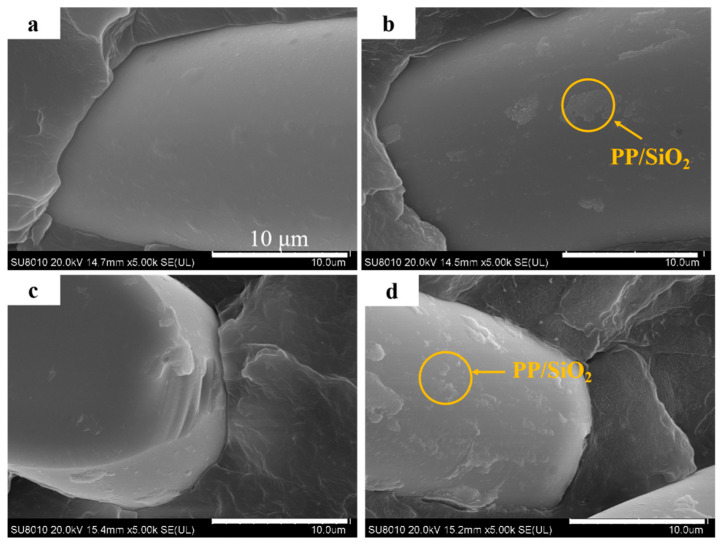
The fracture surface after tensile test, BF (**a**), I_0_BF-gSiO_2_ (**b**), I_100_BF-gSiO_2_ (**c**), I_400_BF-gSiO_2_ (**d**); the white scale bars represent length of 10 μm.

**Figure 10:**
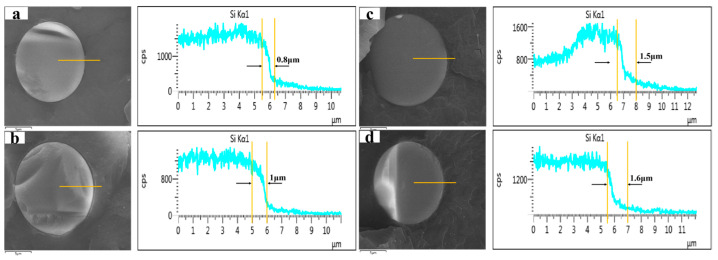
Linear EDS scanning of the interface area, BP (**a**), I_0_BP-gSiO_2_ (**b**), I_400_BP-gSiO_2_ (**c**), I_800_BP-gSiO_2_ (**d**).

The fracture surfaces were then observed using SEM and shown in Figure 9. As can be seen from Figure 9a, the surface of the BF was quite smooth after fracture and the interspace between the fiber and the PP matrix was obvious. When SiO_2_ was grown (Figure 9b), some granules—as the yellow circle indicates—can be observed, which was believed to be the composite of PP and SiO_2_ particles. After irradiation treatment and growth of SiO_2_, the interspace between the fiber and the PP matrix could hardly be spotted, and more residual PP can be observed on the surface of BF; such a phenomenon is quite obvious in Figure 9d. Hence, the evidence from the SEM observation also supported the improved mechanical property of the composites. Furthermore, linear EDS scanning of the interface area was performed. As Figure 10 shows, the thickness of interface layer of BP, I_0_BP-gSiO_2_, I_400_BP-gSiO_2_ and I_800_BP-gSiO_2_ was 0.8 μm, 1.0 μm, 1.5 μm and 1.6 μm, respectively. Therefore, the surface roughening greatly increased the thickness of the interface, which may be the key reason for the improved mechanical property. 

Based on the previous findings, a schematic diagram showing the effect of the SiO_2_ nanoparticles on the processability and microscopic structure of the composites was proposed. During the injection process, the surface-roughed BF acted as a “comb” in a sense, and led to higher shear force and the PP chains being able to be well-aligned. After cooling, the presence of SiO_2_ nanoparticles may contribute to shear-induced orientation and higher crystallinity caused by heterogeneous nucleation. Moreover, the protruding SiO_2_ nanoparticles may form physical interlock with the PP chains and thicken the interface layer, which may be helpful in preventing the chain slippage and conduct the force. Consequently, the reinforcing effect of BF would be better reflected.

### 3.6. Thermal Insulation Properties of the BF/PP Composites

**Figure 11:**
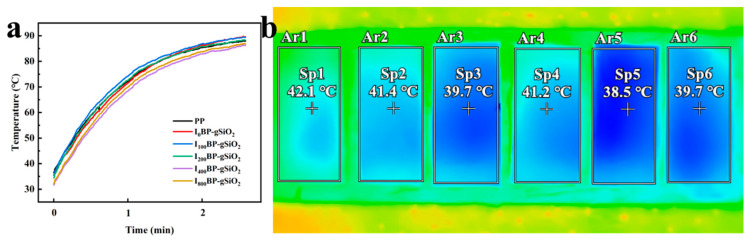
Time–temperature curves (**a**) and the infrared thermal imaging photo of the composites placed at 60 °C (**b**).

The thermal insulation properties of the composites were also evaluated and the results are shown in Figure 11. When placed on a thermostatic graphite plate at 60 °C for 10 s, the surface of PP reached 42.1 °C, while that of I_400_BF-gSiO_2_ was 38.5 °C. And it is obvious that the introduction of modified BF imparts the composites with better thermal insulation properties. It is believed that the good thermal insulation property along with the increased interface may be the key reasons for the improved thermal insulation property of the composites.

## 4. Conclusions

In summary, we proposed a surface roughening technique of basalt fiber by electron beam activation followed by the in situ growth of SiO_2_ nanoparticles, in order to enhance the interaction between the BF and the polymer matrix. The rough surface of BF would lead to stronger shear force and resulted in a higher orientation of the molecular chain while the SiO_2_ could also act as a heterogeneous nucleating agent. As a result, more highly ordered β crystals were generated and lamellar thickness increased. I_400_BF-gSiO_2_ exhibited the highest content of β crystals and showed crystallinity of 53.62% and orientation of β (300) crystal plane of 0.91. Moreover, the protruding SiO_2_ nanoparticles may form a physical interlock with the PP chains and thicken the interface layer, increasing the thermal and mechanical properties of the composites. I_400_BF-gSiO_2_ showed good overall performance, with storage modulus of 8000 MPa at −100 °C, glass transition temperature of 23.03 °C and tensile strength of 62.2 MPa. 

## Figures and Tables

**Table 1:** Key data of the DSC thermograms.

Sample	T_m_ (°C)	FWHM of T_m_ (°C)	T_c_ (°C)	∆H_m_ (J/g)	*X*_c_ (%)
PP	164.99	10.37	107.03	75.88	45.38
BP	164.69	10.65	109.41	75.18	44.96
I_0_BF-gSiO_2_	162.55	9.63	107.14	70.65	42.26
I_100_BF-gSiO_2_	164.88	9.24	111.32	77.48	46.34
I_200_BF-gSiO_2_	165.13	8.93	114.31	80.34	48.05
I_400_BF-gSiO_2_	164.62	8.16	114.17	89.66	53.62
I_800_BF-gSiO_2_	164.46	10.55	114.22	83.77	50.10

**Table 2:** The thickness of long period, lamella thickness and thickness of the amorphous region.

Sample	L (nm)	D_a_ (nm)	D_c_ (nm)
PP	12.68	5.01	7.67
BP	12.92	5.00	7.92
I_100_BF-gSiO_2_	13.02	5.06	7.96
I_400_BF-gSiO_2_	13.27	4.98	8.29

**Table 3:** Key data of the DMA thermograms of BF/PP composites.

Sample	Storage Modulus at −100 °C (MPa)	Storage Modulus at 25 °C (MPa)	T_g_ (°C)
PP	6280	3180	21.80
BP	7768	3905	22.47
I_0_BF-gSiO_2_	7646	4136	22.73
I_100_BF-gSiO_2_	7546	3777	21.94
I_400_BF-gSiO_2_	7909	3642	23.03
I_800_BF-gSiO_2_	7306	3736	20.60

## Data Availability

Data will be made available from the authors on request.
